# A Year Following the Onset of the COVID-19 Pandemic: Existing Challenges and Ways the Food Industry Has Been Impacted

**DOI:** 10.3390/foods10102389

**Published:** 2021-10-09

**Authors:** Márcio Vargas-Ramella, José M. Lorenzo, Benjamin M. Bohrer, Mirian Pateiro, Jesús J. Cantalapiedra, Daniel Franco

**Affiliations:** 1 Centro de Educação Superior da Região Sul-CERES, Universidade do Estado de Santa Catarina, 88790-000 Laguna, Brazil; marcio.ramella@hotmail.com; 2 Centro Tecnológico de la Carne de Galicia, Rúa Galicia No. 4, Parque Tecnológico de Galicia, San Cibrao das Viñas, 32900 Ourense, Spain; mirianpateiro@ceteca.net (M.P.); danielfranco@ceteca.net (D.F.); 3 Área de Tecnología de los Alimentos, Facultad de Ciencias de Ourense, Universidad de Vigo, 32004 Ourense, Spain; 4 Department of Animal Sciences, The Ohio State University, Columbus, OH 43210, USA; bohrer.13@osu.edu; 5 Servicio de Ganadería, Xunta de Galicia, 27071 Lugo, Spain; tsukijizo@gmail.com

**Keywords:** COVID-19, food systems, food safety, food security, public health, risk assessment

## Abstract

The COVID-19 pandemic has created significant impacts for nearly all industrial and societal sectors in the world. As closures and social distancing mandates were implemented to help control the spread of the novel coronavirus designated as Severe Acute Respiratory Syndrome Coronavirus 2 (SARS-CoV-2), the food industry was immensely affected. This review explores the effects of the COVID-19 pandemic on the food supply chain from a multi-disciplinary viewpoint and provides perspectives on the consequences on food safety and food security, a risk assessment on human–animal interactions, and considers logistical/protocol adjustments required for the food industry. While foodborne transmission of the novel coronavirus SARS-CoV-2 is not a significant factor for food safety as direct transmission of the virus through food products is not evident, food security has been significantly affected by the COVID-19 pandemic. The pandemic threatens food accessibility, especially for vulnerable populations of people, through its effects on food cost and infrastructure, food distribution and public transit access, and social inequities. Currently, global interest for COVID-19 is focused on human health and rightfully so, but adverse effects on the food supply chain are already evident and will likely continue to occur for several years after the pandemic is over, let alone if other global health pandemics of this magnitude surface in upcoming years. Uncertainties over the novel coronavirus have interrupted global trade and supply chains. The pandemic has underlined the importance of a robust and resilient food system, which presents an unprecedented challenge for competent authorities in upcoming years.

## 1. Introduction

On 11 March 2020, COVID-19 was declared a global pandemic by the World Health Organization (WHO) [1], and as of July 2021, the pandemic has spread to over 190 million people around the world, leading to more than 4.0 million deaths [2]. Despite the recent devastation that COVID-19 has caused, coronaviruses were first identified as human pathogens in the 1960s [1,3,4]. It is assumed that the novel coronavirus designated as Severe Acute Respiratory Syndrome Coronavirus 2 (SARS-CoV-2, the highly transmissible and pathogenic coronavirus that has caused a pandemic of acute respiratory disease, named COVID-19) crossed the species barrier and infections in humans were initiated through an intermediate host, which could have been either domestic (e.g., pigs, poultry, dogs, or cats) or wild animals (e.g., tigers, lions, ferrets, minks, fruit bats, hamsters, macaques, or snakes) [5,6,7,8].

Food security and food safety are closely linked, and both are a concern during the COVID-19 pandemic. Food security exists when populations of people have physical and economic access to sufficient, safe, and nutritious food that meets their dietary needs and food preferences for a healthy life, while food insecurity exists when populations of people are without reliable access to a sufficient quantity of affordable, safe, and nutritious food [9]. Whereas food safety refers to handling, preparation, and storage of food with conditions or practices that reduce the risk of individuals becoming sick from foodborne illnesses [10,11,12]. Foodborne transmission of SARS-CoV-2 is not supported by scientific evidence which relieves food safety risks that could be caused by direct contamination [6,13,14,15,16], yet food security was, and continues to be, significantly affected by the COVID-19 pandemic. The pandemic threatened food accessibility through its effects on food cost and infrastructure, food distribution and public transit access, and social inequities; all of which contributed to shortages of some food products and the inability for some individuals to procure enough food products to meet their nutritional requirements [17]. For instance, many countries throughout the world announced (and imposed) restrictions for food processing plants in the form of slow-downs, shut-downs, and even closures in response to COVID-19 infections among workers [18,19,20].

Currently, global interest for COVID-19 is focused on human health, and rightfully so, but adverse effects on the food supply chain are already evident and will likely continue to occur for several years after the pandemic is over [6]. Additionally, reports show a clear change in consumer behavior and purchasing habits for food (e.g., greater preference for shelf-stable food products and lower preference for perishable food products). These changes are likely caused by more than just greater at-home consumption and less food-service consumption [21]. The demand for high-value, safe food products as well as other factors such as social class (high/low-income families) and age (adolescents, young adults, or older adults) are influencing food preferences [1,10]. Moreover, restrictions caused by the COVID-19 pandemic, which require people to stay at home and socially distance, provided people more time to prepare healthy meals at home, but also opportunities to become bored or anxious, which likely increased unhealthy snacking, the use of alcohol, and the frequency of smoking [14,17,21,22,23,24,25]. The ongoing COVID-19 pandemic caused a global downturn comparable to that of the great depression in the 1930s [26,27]. Uncertainties over the novel coronavirus have interrupted global trade and supply chains [28]. The pandemic has underlined the importance of a robust and resilient food system [29], which presents an unprecedented challenge for government authorities [30]. Consequently, this review explores the effects of the COVID-19 pandemic on the food supply chain from a multidisciplinary viewpoint and includes a specific focus on the consequences of COVID-19 on food safety and food security, a risk assessment on human–animal interactions, and the logistical/protocol adjustments required for the food industry.

## 2. COVID-19: Consequences for Food Safety and Food Security

Before the pandemic, the WHO estimated there were 600 million cases of foodborne diseases associated with contaminated foods each year, resulting in 420,000 deaths [31]. Even so, public health issues caused by food safety crises can change consumers’ beliefs [10]. For instance, the perception of a food crisis in Europe (e.g., Bovine Spongiform Encephalopathy-BSE, dioxins, and Polychlorinated Biphenyls-PCBs) has generated suspicions among consumers about the safety of food products procured from conventional livestock production, which has, in turn, generated incremental increases in the demand for organic products procured from livestock raised using “traditional” practices (e.g., raised without the use of antibiotics and other conventional technologies or methods) [32,33]. However, it should be noted that currently there is no scientifically-based evidence to suggest that food can be a vector for the transmission of SARS-CoV-2, thus significantly reducing food safety concerns and suspicion from consumers [29,34].

As the COVID-19 pandemic progressed, the implementation of mandatory isolation and social distancing regulations (i.e., restrictions, lockdowns, curfews, quarantines, etc.), that were required to contain and limit the spread of the virus, has created a food security crisis [19]. Food security is a crucial issue that should be carefully considered during the implementation of mandatory isolation regulations for the public and before restrictions are mandated in the food industry [1,14,23]. In the past, public health pandemics (e.g., avian flu and African swine fever) directly impacted the meat industry causing a reduction in the production of meat products. Similarly, the COVID-19 pandemic has directly and severely impacted food production and, in turn, food security. The four pillars of food security, which are (1) availability (food supply), (2) access (necessity vs. food availability), (3) utilization (enough intake of required nutrients), and (4) stability (consistent access to food) have all been affected by the COVID-19 pandemic [19].

### 2.1. COVID-19 Pandemic: Food Security vs. Dietary Behavior

As already mentioned, the COVID-19 pandemic has modified the consumption patterns and dietary behaviors of people. For instance, individuals with obesity have experienced more severe clinical SARS-CoV-2 symptoms and have a greater risk of mortality once infected [14]. The pandemic also raised awareness in people about the importance of strengthening the immune system through healthy eating. The establishment of healthy dietary habits is crucial to support the human immune system and, if sustained over long periods of time, could have a positive impact in preventing SARS-CoV-2-related complications [14,23]. In line with this, diets high in carotenoids, flavonoids, and vitamin A (such as diets with high amounts of carrots, spinach, and sweet potato) [1,35], diets high in vitamin C (such as diets with high amounts of citrus fruits, kiwifruits, and broccoli) [1,36,37], and diets high in vitamin D [21] have been shown to improve immune function. Indeed, a recent study has suggested that adequate supplementation with vitamin C, vitamin D, and vitamin E may enhance resistance to severe SARS-CoV-2 symptoms, although it is important to highlight that diet recommendations to improve the immune system should be interpreted with caution, because other factors such as personal hygiene, adequate levels of exercise and physical activity, adequate nutritional intake, age, pre-existing health conditions, and general lifestyle play a key role in immune system function [38]. Concerning vitamin D, some studies have suggested that food supplements containing vitamin D at very high levels can protect individuals against infection of SARS-CoV-2 [39]. However, these assumptions are based on a small number of publications reporting that SARS-CoV-2 patients often have insufficient serum levels of vitamin D, hence despite the scientific consensus regarding the role vitamin D plays in the immune system, there are still many unanswered questions about its efficacy against SARS-CoV-2 infection [40].

During the pandemic period, it has been reported that the willingness of individuals to adopt healthier habits depends on the country, social class, and food product [17,19]. For instance, in Spain the adult population has incorporated healthier dietary behaviors, based on the principles of the Mediterranean diet (i.e., vegetables, fruits, herbs, nuts, beans, and whole grains), thus decreasing consumption of fried foods, snacks, fast foods, red meat, pastries, and sweet beverages [23]. On the contrary, an opposite trend was observed in Poland with a decrease in the consumption of fruits, legumes, and vegetables, while snacking, alcoholic beverage consumption, and the use of cigarettes increased [14]. Regarding social class, the pandemic has made it clear that limited access to services for marginalized sections of the population causes many individuals to have limited access to fresh, healthy, and affordable food [21]. Certainly, current situations for vulnerable populations were exacerbated by the COVID-19 pandemic (e.g., school closures, less free food delivery services, business closures, and job losses), and social inequities have increased to levels well beyond those seen before [19,21,27]. Unfortunately, the economic projections have become increasingly pessimistic in relation to food security in the coming months. Indeed, the International Monetary Fund has estimated in 2020 a 5% decline in the world economy [41], which is a deeper global recession than the global financial crisis of 2008–2009 [19].

Last but not least, historical and socio-cultural factors have significant relevance to the COVID-19 health pandemic. To illustrate this fact, it should be noted that Southeast Asia has been identified as a “wildlife trade hotspot” for many years (dating back to prehistoric times in China), where wild game meat is perceived to have high medicinal value based on superstitions and philosophical reasons [10,42,43]. In China for example, pangolin meat is believed to help relieve rheumatism, promote blood circulation, eliminate irascibility, and improve eyesight, among several other health-promoting effects [44]. These cultural factors make the widespread implementation of new food safety systems challenging.

### 2.2. The Transmission of SARS-CoV-2 through Oral/Alimentary Routes

The transmission of SARS-CoV-2 can be predominantly defined by person-to-person interaction (absorbed by the mucous membranes of the respiratory tract, eyes, nose, or mouth), particularly via droplets, and by close contact with infected individuals [1,3,5,18,42,45,46]. Although, numerous other forms of direct and indirect (fomite) transmission can occur [5,13]. Transmission modes of SARS-CoV-2 include respiratory droplets (>5–10 μm in diameter), airborne transmission (droplet nuclei or aerosols with < 5 μm in diameter suspended in air), fomite particle transmission (contaminated surfaces), fecal-oral particle transmission, blood-borne transmission (low risk), mother-to-child transmission (no evidence for intrauterine transmission), or animal-to-human transmission [45,47,48]. In addition, SARS-CoV-2 can be transmitted to healthy individuals by symptomatic, pre-symptomatic (thought to be a major contributor), and asymptomatic patients [3,5]. Several reports of widespread outbreak have been related to crowded and poorly ventilated indoor spaces, which suggests the possibility of aerosol transmission in addition to droplet transmission [3,49,50]. Indeed, aerosol transmission may be a more important exposure transmission pathway than previously considered. Humans produce aerosol particles continuously through normal respiration, and aerosol production increases during respiratory illnesses [51]. In addition, viral load in the upper respiratory tract appears to peak around the time of symptom onset and viral shedding begins approximately two to three days prior to the onset of symptoms [3].

Alternatively, indirect transmission (fomite) may also occur through respiratory droplets that could land on immediate surfaces. Thus, an individual may become infected by touching their own face, mouth, nose, or eyes following contact with high-touch surfaces (e.g., handrails, doorknobs, or working tools) [1,13]. Fomite transmission is plausible since the virus can remain viable and infectious on surfaces up to several days after contamination [52]. Nonetheless, the clinical significance of SARS-CoV-2 transmission from surfaces is difficult to interpret without knowledge of the minimum dose of virus particles required to cause infection [3]. By now, there is no scientific evidence that has directly demonstrated fomite transmission. Individuals who encounter potentially infectious surfaces usually have close contact with an infectious person, hence the distinction between respiratory droplet and fomite transmission is difficult to discern [47].

Regarding the oral/alimentary pathway-although coronavirus may reach fresh food products or food packaging through an infected person who is sneezing or coughing directly on them, foods are not a direct source for the transmission of SARS-Cov-2 [6,16]. It is highly unlikely that individuals can contract SARS-CoV-2 via food or food packaging [48,53]. This fact was also confirmed in the past as studies conducted with previous coronaviruses (SARS-CoV and MERS-CoV) showed that oral/alimentary transmission, via the esophagus and stomach, did not occur [1,6,42]. In addition, oral transmission of virus-containing foods is protected by the acidic conditions of the stomach (pH < 3.5), which would inactivate SARS-CoV-2 [6]. At the same time, it should be noted that this route is theoretically possible in individuals with reduced gastric acidity due to drugs or specific medications [45]. Despite the evidence, the food industry is not excluded from extensive COVID-19 prevention programs. Within the “farm-to-fork” concept of food production, many safety measures are required since several potentially high-risk sources of infection are involved in the production of food [6]. Therefore, it is imperative for the food industry to reinforce personal hygiene, physical distancing measures, and provide refresher training on food hygiene principles for food workers [13,15,16] as well as develop analytical protocols for food and environmental safety [6].

### 2.3. Animal Origin Products: The Risks of Consumption

Despite the working hypothesis that SARS-CoV-2 may have originated in bats and infected other animals before infecting humans through consumption of animal-derived food products, there is no evidence of continued transmission of the virus from animals to humans through the food chain [16,46]. Based on currently available information, no additional COVID-19 related sanitary measures need to be applied during the production of animal-derived food products. These measures, which are already implemented prior to the COVID-19 pandemic, comprise of good hygiene practices during food handling and preparation to avoid potential cross-contamination [1,16]. The consumption of cooked meat (of domestic or wild origin), eggs, and milk from healthy livestock is not considered a risk factor for acquiring infection from SARS-CoV-2 [40,54]. Considering that coronaviruses are thermolabile, the normal cooking temperatures of meat and eggs (i.e., 70 °C) or the pasteurization process for milk (i.e., 63 °C for at least 30 min) are effective methods to inactivate the virus [16]. In addition, it has been proven that the transmission of SARS-CoV-2 via milk is unlikely since coronaviruses cannot be transmitted from cows fed with contaminated feed [40]. An infection of consumers with SARS-Cov-2 only appears theoretically possible if food is touched shortly after contamination and the virus is then transmitted indirectly to mucous membranes. Even so, contamination of foods with other bacterial pathogens (e.g., *Salmonella* spp., *Campylobacter* spp., *Escherichia coli*, and *Listeria monocytogenes*) still represents the most significant global food safety concern [16].

## 3. Live Animals: The Risks of SARS-Cov-2 Transmission to Humans

Regarding live animals, several different coronaviruses that affect humans have natural reservoirs, such as bats, camels, civets, and pangolins [1,3,4]. In the case of SARS-Cov-2, it is thought that the virus first infected humans through an intermediate host which could have been a domestic or wild animal [5,6,7,46,47,55,56]. For this reason, events with human–animal interactions (e.g., zoos, animal trading fairs, and livestock exhibition events) might also come into question as they may lead to an increase in the size of the pathogen reservoir [42]. In addition, the most suspected reservoir hosts are those animals that are highly stressed or particularly ill (although the virus does not necessarily cause clinical illness) [42].

In laboratory conditions, the incubation period in animals appears to be similar to humans (i.e., in the range of 2–14 days with an average duration of 5 days after infection) [1,3]. Current evidence suggests that clinical signs may include coughing, sneezing, respiratory distress, nasal discharge, ocular discharge, vomiting, diarrhea, fever, and lethargy, but as in humans, asymptomatic infections also occur in animals [56].

### 3.1. Animal Species Susceptible to Infection with SARS-CoV-2

Findings from laboratory studies suggest that cats and ferrets are the most susceptible animal species to infection with SARS-CoV-2, but many other species have proven to be susceptible either through natural infections or by experimental infection (Table 1) [5,46,54,56]. Furthermore, ferrets were susceptible to SARS-CoV-2 infection under laboratory conditions and were able to transmit the virus to other individuals, which is useful for vaccine or therapeutic research [54].

Experiences with previous outbreaks of SARS-CoV and MERS-CoV have demonstrated that the virus is usually transmitted through intermediate hosts (civets and camels, respectively) before transfer to humans occurred [1,3,4,5,21,42,57]. Although several animal species have been infected with SARS-CoV-2 in multiple countries, many of these infections are not considered as drivers of the COVID-19 pandemic [16,54,56].

Several reports have shown that carnivore species (e.g., cats, dogs, tigers, lions, and minks) could be naturally infected with SARS-CoV-2 [46,55]. On the contrary, there is no evidence that SARS-CoV-2 could be transmitted to humans by domestic food-producing animals (e.g., poultry, pigs, cattle, camels, horses, sheep, goats, rabbits, guinea pigs, and fish) [16]. Therefore, extensive quarantine measures (beyond normal pre-COVID-19 protocols) are not necessary for livestock species, even if animals are imported from countries experiencing high levels of SARS-Cov-2 infection rates [40,48,53,58]. Further studies are needed to understand how different species could be affected since information about routes of transmission among animals is still limited [50,59].

### 3.2. Humans and Animals with Close Contact: Possible Routes of Transmission and Precautionary Measures for SARS-CoV-2

**Figure 1:**
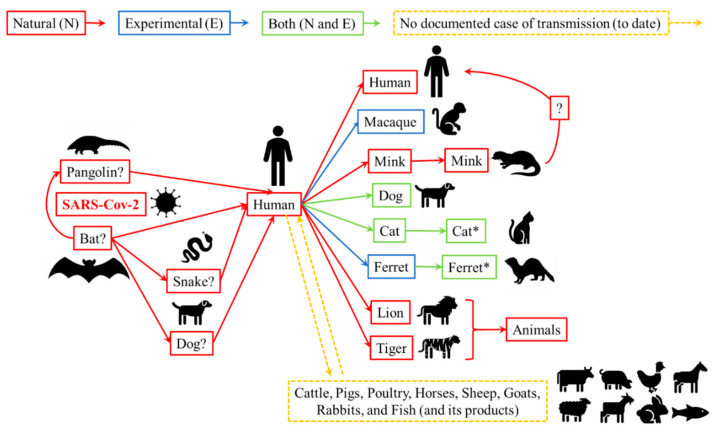
The human–animal transmission route and host range of SARS-CoV-2 occurred naturally and/or experimentally: suggested (not confirmed source and/or route); * animals kept together with experimentally infected ones. Adapted from Houssain et al. [5], Newman et al. [46], and OIE [56].

Individuals who are suspected or confirmed to be infected with SARS-CoV-2 should avoid or minimize direct contact with animals [5,46,55]. Several studies suggest that a diversified route for human–animal transmission and host range could occur (Figure 1). To date, there are no documented cases of direct bat-to-human transmission, hence other wildlife species may be involved as the intermediate hosts between bats and humans [16]. On the other hand, carnivore species, especially domestic pets like dogs and cats, may be a more plausible reservoir of SARS-CoV-2. According to several studies, pets could be infected from their owners, from minks, from other wild animal species, or even from caretakers of tigers and lions [5,46]. Despite the possibility of spreading SARS-CoV-2 through domestic pets [5], there is no evidence that pets are playing a key epidemiological role in the spread of SARS-CoV-2, thus there is no justification to take measures directed towards pets which may compromise their welfare or well-being [54,60].

Nevertheless, precautionary measures with pets belonging to owners infected (or potentially infected) with SARS-CoV-2 should be contemplated. Pets should adhere to similar social distancing and quarantine guidelines as humans (i.e., remain indoors when possible, avoidance of close contact (>2 m) if possible, and quarantine if experiencing SARS-CoV-2 symptoms) [54,60]. Testing is only recommended for companion animals when typical SARS-CoV-2 symptoms are experienced and when exposure occurs with a person infected with SARS-CoV-2 [5,46,55]. Similar to humans, quarantine restrictions can end for pets once the animal has not shown clinical signs of SARS-CoV-2 for 72 h, and 14 days have elapsed since their last positive test [60].

## 4. Food Industry: Steps to Prevent SARS-CoV-2 Contamination (Human, Surfaces, and Food)

Food safety is a scientific discipline that studies hazardous factors related to animal disease, microbiological safety, and several other factors related to consumer health [10]. In order for the food supply chain to remain safe from virus contamination, the main priority from a food safety standpoint is to keep the virus out of the food environment. In addition to the cleaning and sanitation measures normally applied in the food industry, educating staff on additional COVID-19 protocols such as physical distancing, facial coverings (i.e., masks and shields), and extra hand washing is of critical importance [40,53]. Different precautions are recommended for each stage within the “farm-to-fork” process, although precautions are usually most necessary at the final stages of the process (i.e., point of consumer purchase, in-home preparation, and/or consumption) where more people (potential sources of infection) and more uncontrolled external environmental factors are involved [6].

Globally, slaughterhouses and meat processing facilities have been a major risk for the spread of SARS-CoV-2 infection throughout the COVID-19 pandemic [20]. It can be partially explained by the favorable environments for SARS-CoV-2 transmission. This includes environmental factors inherent to slaughterhouses and meat processing facilities like the high number of metallic surfaces, low temperatures, and high relative humidity, as well as the dense production of aerosols from workers and the intense use of water for various events through the production chain, such as carcass washing, that could transport virus particles [18,20,61]. Additionally, in some production facilities, workers must speak loudly over background noise (releasing virus droplets), workplaces are crowded for long periods of time, many high-touch surfaces exist, social distancing is difficult to maintain due to assembly-line processes, and finally, the age of workers is relatively low and therefore workers are prone to have asymptomatic infections [20].

### 4.1. Food Inspection Services (Government Agencies): Global Trading (Importing and Exporting)

As food products have not been implicated in the direct transmission of SARS-CoV-2, imported foods from countries with a high prevalence of SARS-CoV-2 infections should have the same import controls and regulations as those used before the COVID-19 pandemic [40,58]. However, the pandemic has affected the trade of most goods through additional border control, lack of cargo shipments, and greater reinforcement of sanitary controls. Indeed, some countries have imposed export restrictions in order to protect their domestic consumers. For example, twenty-one countries announced export restrictions covering globally traded food products at the beginning of the COVID-19 pandemic [19,26].

In addition, and as stated by the WHO, food inspectors routinely wear protective equipment and there is generally no need for additional protective equipment beyond that of special COVID-19 equipment like facial coverings (i.e., mask or shield) and special COVID-19 guidelines like social distancing [58]. With that said, the government agencies of several of the leading meat-producing countries such as Argentina [62,63], Brazil [64,65], India [66], and the USA [67] updated their legislation on protective equipment and published additional guidelines in order to prevent, control, and mitigate SARS-CoV-2 infection rates in the food processing industry. Furthermore, according to the WHO [58], food safety authorities should also consider reducing the frequency and requirements of food inspectors during the COVID-19 pandemic in order to limit the spread of SARS-COV-2 infections.

### 4.2. Personal Hygiene of Food Workers

Before the pandemic, all food processing facilities should have had dedicated areas at the entrance of food workspaces with warm running water, liquid soap, hand sanitizer, tissues/towels, and closed waste bins. Now, additional services in crowded areas in the workspace should be considered. Moreover, all employees must be trained and provided with the proper PPE (Personal Protective Equipment) for the work-related tasks such as gloves, eyewear (glasses or goggles), gowns, aprons, and hardhats, but additional PPE may be required due to the COVID-19 pandemic including medical masks or face shields. In addition, good hygienic practices among workers should include good respiratory hygiene (i.e., covering the mouth and the nose when coughing or sneezing, and properly disposing of used tissues) [48].

Protocols for personal hygiene recommended before the COVID-19 pandemic could be maintained despite the ongoing and additional measures related to COVID-19 because these measures regularly proposed for food processing plants are effective in preventing the spread of other viruses such as influenza. However, it should still be a priority to inform food workers of the need to thoroughly and frequently wash and sanitize their hands [16,48]. Indeed, handwashing with normal soap and warm running water has already been proven to be a greater protective barrier to infection than wearing disposable gloves [48,68]. In this regard, it is important to highlight that disposable gloves can give a false sense of safety and should not be used in the working environment of food production facilities as a substitute for handwashing. Food workers should not touch their faces or contaminated surfaces when wearing gloves. The SARS-CoV-2 virus can contaminate disposable gloves in the same way it contaminates the hands of workers and contact surfaces [53]. Finally, communication materials on personal hygiene guidelines must be published with instructions on how to apply recommendations correctly.

### 4.3. Cleaning the Environment and Facilities

In order to reduce the risk of infection through fomites, it is essential to establish procedures for the correct disinfection of environments (i.e., air, water, and surfaces) and facilities [47]. Similar to the already discussed section on personal hygiene, cleaning and sanitizing programs routinely used by the food industry before the COVID-19 pandemic are also effective against SARS-CoV-2. For most cleaning protocols, water can be used since standard treatment methods are sufficient to inactivate the virus [69]. Similar to the already discussed section on personal hygiene, cleaning and sanitizing programs routinely used by the food industry before the COVID-19 pandemic are also effective against SARS-CoV-2. For most cleaning protocols, water can be used since standard treatment methods are sufficient to inactivate the virus [16]. As enveloped viruses, coronaviruses react sensitively to substances that dissolve fat, such as alcohols or surface-active agents, which are contained in soaps and detergents as grease removers [40]. However, it is recommended to increase the frequency of cleaning procedures especially for high-contact spaces and surfaces (e.g., keyboards, handrails, door handles, elevator buttons, tables, and chairs). Natural ventilation should be prioritized and, if possible, air exhaust fans should be set to full power during sanitization [64,65]. It is important to follow manufacturers’ recommendations regarding: (1) disinfectant use, notably the need to first remove organic matter that can inhibit contact and neutralize the efficacy of disinfectants; (2) dilution of the disinfectant; and (3) the contact time required to be effective [16]. The most recommended disinfectants to inactivate coronaviruses according to different references are shown in Table 2.

Previous studies that measured the survival time of SARS-CoV-2 in the environment suggested that the virus is resilient to aerosols. The authors found that aerosol suspension with SARS-CoV-2 persisted up to 16 h, which was longer than what was expected [51]. Therefore, if cleaning is required in a specific location where a suspected or confirmed case of COVID-19 was identified, the location should be first well ventilated with fresh air for at least one hour, and then cleaned with a detergent followed by disinfectant decontamination [47]. Overall, SARS-CoV-2 can be highly stable in a favorable environment, but it is also susceptible to standard disinfection methods [70].

It is important to highlight that despite laboratory tests that have shown coronaviruses can remain infectious on several different surfaces and in several different environments, these findings were from controlled experiments, hence they should be interpreted with caution. In line with this, a significant risk of SARS-CoV-2 transmission by fomites has been assumed on the basis of research that has little resemblance to real-life scenarios [72]. In agreement with this, Wiersinga et al. [3] suggested that the clinical significance of transmission from fomites is difficult to interpret without knowing the minimum dose of virus particles that can initiate an infection. Previous studies have found the longest survival period (2–6 days) of coronaviruses by placing a very large initial virus titer (10^4^ × 10^7^ infectious virus particles) on the surface being tested [52,73,74], but none of these studies present scenarios similar to real-life situations [72]. Indeed, Goldman [72] published that the chance of transmission through fomites is limited and it could happen only in situations where an infected person coughs or sneezes on the surface and someone else touches that surface soon after (within 1–2 h).

### 4.4. Coronaviruses (SARS-CoV): Minimum Conditions to Environment Survival and Methods for Inactivation

The stability of coronaviruses in different environments (Table 3), as well as the period that viable virus and/or Ribonucleic Acid (RNA), can be found by Reverse Transcription Polymerase Chain Reaction (RT-PCR) depends on several factors, including the virus strain and quantity [50].

Recent studies [13,45,70] have demonstrated that SARS-CoV-2 may remain infectious on surfaces in ambient temperature (20–37 °C) for up to 72 h, but most virus particles become inactive (noninfectious) after the first 24 h. However, it is important to emphasize that this survival period can greatly increase (>14 days) on contaminated surfaces, especially in fluid suspensions, and at lower temperatures [1,40,45,70].

Finally, despite the fact that SARS-CoV-2 seems to be stable on smooth surfaces (e.g., glass, paper, stainless steel, and plastic) and in a wide range of pH values at room temperature (pH 4–10) [45,70], researchers have recently published that this virus is considered less stable than many other pathogens [40]. To date, however, very little is known about how this virus survives outside the human body [13,53]. Similar to the already discussed section on environmental cleaning, studies for virus environment survival should be interpreted with caution since data were obtained from experiments that do not represent real-life situations [3,72].

### 4.5. Processing Facilities: Food Industry Challenges to Prevent and Control COVID-19

During a pandemic, authorities and the research community should quickly identify the most critical threats to the food system in order to implement mitigation measures [1]. Despite the fact that SARS-Cov-2 transmission through the food sector is considered negligible, the transmission in the food supply chain may be possible from infected workers and the surrounding environment [6]. Considering this, developing guidance to prevent and control SARS-Cov-2 transmission in working facilities is required to ensure that workers are able to perform their duties in an environment with enhanced safety measures.

Food processing facilities should consider the type of food being processed or handled, methods and scale of the operation, possible at-risk groups among workers and consumers, the history of compliance or non-compliance with food legislation, and the distribution channels for the food products [30]. For instance, the USA Centers for Disease Control and Prevention (CDC) [18,77] congregated data from facility risk assessments among meat processing plants and their workers regarding SARS-CoV-2 infection. As a result of this information, guidance was developed with facilities’ challenges in mind to prevent and control the virus. The challenges were divided into three categories: structural, operational, and sociocultural. The challenges according to practices and sectors with their recommended suggestions in the facilities are shown in Table 4.

The congregate dataset allowed the food supply chain to identify and control food safety hazards according to the risk profile of each type of processor. Consequently, data obtained about critical threats, risk assessments, and challenges during the pandemic should be used by the food industry to elaborate and maintain management systems and food safety guides. There are potential hazards at every stage of the food production process [78], but mitigating risk should be a priority.

A management system is the way in which an organization manages the interrelated parts of its business in order to achieve its objectives, which can be related to food safety [79]. For instance, congregate data obtained about risk assessment can provide a model to follow for the implementation of a Food Safety Management System (FSMS) [80]. Additionally, FSMS should be based on the Hazard Analysis and Critical Control Point (HACCP) principles in place to manage food safety risks and prevent food contamination [78]. The food industry FSMS is also underpinned by prerequisite programs that may include, but are not limited to, the ISO 9000 series, ISO 2200 series [48], and Good Manufacturing Practices (cGMPs) [81]. Notwithstanding, the food industry challenges in relation to the COVID-19 pandemic warranted the revision of management systems of food industries, including most quality programs such as HACCP.

HACCP is universally recognized as a very effective way to mitigate the risks in the food industry. Implementation of a successful HACCP plan promotes a systematic preventive approach to improve food safety in light of the biological, chemical, and physical hazards [78]. Generic HACCP plans can serve as important guides in the development of the plan, but the format of the plan is product- and process-specific. The information obtained during the process of HACCP implementation or maintenance, such as the facility risk assessments, is essential and required for hazard analysis. A thorough hazard analysis is the key to preparing an effective HACCP plan and FSMS in a food processing plant [81].

## 5. From Farm-to-Fork: Protocols to Identify and/or Prevent SARS-Cov-2 on (or in) Foods

As food has not been implicated in the transmission of SARS-CoV-2, continuous testing of food or food surfaces for SARS-CoV-2 is not necessary [58]. In relation to the detection of the virus in environmental samples, several studies have been carried out with environmental swabs and kits. These results are preliminary and were rather expensive to generate [6], hence the accurate and cheaper diagnosis of SARS-Cov-2 in the food chain is an ongoing challenge [82].

### 5.1. Detection Tools for SARS-CoV-2 in Workers and Facilities

The most used diagnostic tools for SARS-CoV-2 infections are based on viral gene detection (RT-PCR), serological tests (ELISA), and radiological findings [computed tomography (CT), X-ray, and Ultrasound] [82,83,84,85]. Moreover, there are various analytical methods used for the confirmation of SARS-CoV-2 infection. Table 5 summarizes several methods for SARS-CoV-2 detection according to samples and laboratory methods.

The aforementioned tools were developed for human patients, hence the detection of SARS-CoV-2 in food, surfaces, and surrounding environments could be more challenging than the human test [6]. The viral contamination of the food supply chain environment (i.e., air, water, and surfaces) could have serious implications for COVID-19 control strategies. For this reason, in addition to individual diagnosis, sampling the air surfaces surrounding environmental facilities could represent an auxiliary tool to evaluate if workers operate with adequate safety. Furthermore, SARS-CoV-2 detection could assess the efficacy of the measures of guidance developed to prevent and control SARS-CoV-2 transmission in processing plants.

With regard to human testing, SARS-CoV-2 gene detection made possible via RT- PCR is considered the “gold-standard” test and is the primary method for COVID-19 diagnosis [3,38,82,84,85,86]. However, this protocol has limited application for use as a routine test because of its technical complexity [82,83] and false-negative results (which may occur for up to 67% of patients) [3,86]. Despite this, nucleic acid amplification tests (NAAT) are the most sensitive and specific assays and are generally the preferred test to detect early viral infections. The different types of NAAT assays, such as RT-qPCR, RT-LAMP, microarray, and high-throughput sequencing have been developed for the rapid and accurate diagnosis of COVID-19 [82]. Viral nucleic acid (not viable virus) can be detectable in throat swabs for up to six weeks after the onset of illness while viral cultures are generally negative eight days after the onset of symptoms [3].

Concerning serological tests (e.g., ELISA), detecting the antibodies produced by the immune system in response to the viral infection is one of the most important techniques. ELISA is an established serological test in detecting SARS-CoV-2 active cases due to how the viral RNA becomes almost undetectable at 14 days post-illness. The antigen-specific antibody can be detected in a patient after three to six days following the infection [82]. However, false-negative results may also arise since most patients could develop an antibody after approximately 14 days in response to SARS-CoV-2. In addition, the biggest issue with this test is the cross-reactivity that can lead to false positives [85].

Concerning radiological findings, typical CT imaging, with ground-glass opacities associated with the number of lung segments involved, was found to be related to disease severity [83,84]. Moreover, CT scans have a higher sensitivity (up to 98%), yet the specificity (25%) of this test is low and could lead to a high percentage of false-positive results [82,84]. Conversely, conventional chest X-ray sensitivity is low (~59%) and ultrasound has been used as a diagnostic tool in a very limited number of cases due to the low specificity. However, ultrasounds are important to monitor the progression of the disease. CT and ultrasound findings appear to be superimposable, but at the current time, the best radiological strategy remains undefined [83].

Diagnosis with laboratory detection and radiographic imaging is not always in agreement with the clinical features of patients [86]. Current studies suggest that the risk of diagnostic errors could be minimized following some practical recommendations: (1) combining clinical evidence with results from chest CT and RT-PCR; (2) RT-PCR results interpreted according to epidemiological, clinical, and radiological factors; and (3) upper (or lower) respiratory tract specimens re-tested in patients with negative RT-PCR and high suspicion of infection [82,83].

In relation to the environment (i.e., air, surfaces, and water), air sampling can be performed using commercial bioaerosol samplers placed in the ambient environment to collect particles. Regarding surfaces, sampling can be measured with pre-moistened macrofoam sterile swabs. Yet, samples need to be immediately stored at 4 °C or −80 °C if not immediately analyzed. Then, RNA extraction is processed to perform real-time PCR assays [87]. On the other hand, wastewater monitoring has been a successful strategy pursued to track viral diseases by the detection of genetic material. Therefore, this tool is recommended to be implemented in wastewater treatment plants as a tool designed to help authorities coordinate social distancing regulations (i.e., restrictions, lockdowns, curfews, quarantines, etc.). To apply this method, viral RNA is extracted from concentrates with commercial kits from water samples and analyzed with real-time quantitative PCR (RT-qPCR) [88].

The COVID-19 pandemic has proven the worth of rapid diagnostics. Prompt and accurate testing of SARS-CoV-2 infections is still an emergency as the virus is continuing to spread [85]. In early 2020, WHO published that the development of a rapid and accurate nucleic acid and protein test with detection at the point-of-care is a priority [89]. To date, many of the published rapid diagnostic methods have relied on field-effect transistors (FET) and surface plasmon resonance (SPR) principles, which are known to deliver fast and sensitive signals [85]. Concerning emerging diagnostics for SARS-CoV-2, nucleic acid tests using isothermal amplification (e.g., LAMP assay) are currently in development for SARS-CoV-2 detection. This technique is advantageous since it does not require specialized laboratory equipment to provide similar analytical sensitivities to PCR [84,85]. On the other hand, rapid serological tests (e.g., blood tests for antibodies) can be particularly useful, but drawbacks include potential cross-reactivity of SARS-CoV-2 and false-positive results. Finally, diagnostic test manufacturers also should focus on the development of self-testing home kits to be able to see the real extent and progress of COVID-19 worldwide and act accordingly [85].

### 5.2. Coronavirus Disease (COVID-19): Protocol to Employee Illness Working in the Food Industry

Work procedures employed by food companies as part of their FSMS should ensure that infected workers and their contacts are excluded from food processing facilities. A procedure to allow staff to report illness by phone (or email) should be established with the aim that workers can receive reliable information during the early stages of a SARS-CoV-2 infection [48,65]. If a worker becomes ill, the employee should keep a distance of at least 2 m from other individuals or be removed to an isolated area with an open window for ventilation [48,53]. All surfaces that the infected employee has come into contact with must be properly cleaned and sanitized (Table 2).

According to current guidelines for the diagnosis of COVID-19, confirmed patients could be released from isolation once their symptoms resolve and they have two negative PCR tests with an interval of at least 24 h [48,53,83,86]. If testing is not possible, WHO recommends that a confirmed patient can be released from isolation 14 days after symptoms resolve [48,53,71]. The difference between quarantine and isolation should be noted and understood. Quarantine means “the restriction of activities and/or separation from others in such a manner as to prevent the possible spread of infection or contamination, and this applies to both suspect persons who are not ill and to suspect baggage, containers, conveyances, or goods” [90]. Quarantine is different from isolation, which can be defined as “the separation of infected persons from others to prevent the spread of the virus” [71].

Individuals could be considered ‘contacts’ if they are in contact with an infected person (i.e., within 1 m for at least 15 min from 2 days before illness onset, or 2 days prior to positive sample collection for asymptomatic patients, and up to 14 days after an infected person developed symptoms or laboratory confirmation) [3,49,65,71,91,92,93,94,95,96]. Contacts should self-quarantine and self-monitor (i.e., report symptoms and record temperature twice a day) for 14 days from their last exposure (i.e., close encounter with confirmed or probable COVID-19 case) [71,94,95,96]. Examples of contacts with a confirmed case in the food industry could include face-to-face or physical (i.e., touching) contact, any employee who was within one meter of another person, anyone who has cleaned up any bodily fluids without adequate PPE (e.g., gloves, overalls, or other protective clothing), employees in the same working team or workgroup, and any employee living in the same household [48,53,65].

For contacts who do not develop symptoms, WHO no longer considers laboratory testing a requirement for leaving quarantine after 14 days of the quarantine [71]. It appears that transmission is possible for approximately eight days after symptoms appear [83]. Prolonged PCR test positivity (>8 days after infection) probably does not correlate with clinical transmission [83,84]. However, considering the probability of false-negative results, recommendations that define the length of isolation for patients should be considered with caution.

Taking the temperature of food workers is not recommended as the only control measure to avoid infection, fever is only one of the symptoms of COVID-19, and absence of fever alone is not a reliable indicator of wellness [48,53]. Common symptoms in hospitalized patients include fever (70–90%), dry cough (60–86%), shortness of breath (53–80%), fatigue (38%), myalgias (15–44%), nausea and/or vomiting or diarrhea (3–39%), headache, weakness (25%), and rhinorrhea (7%) [3,86]. The symptoms expressed by COVID-19 patients are nonspecific and cannot be used for an accurate diagnosis [84].

Finally, according to Jones et al. [92], despite physical distancing being an important part of the measures used to control COVID-19, the 1 to 2 m distancing guideline used for physical distance protocols to reduce transmission of SARS-CoV-2 is based on an outdated notion, that began in the 19th century. Further work is needed to extend a fully effective social distancing guideline that works in all conditions (e.g., indoor vs. outdoor settings, different ventilation conditions, different rates of occupancy, exposure time, host viral load, duration of exposure, number of infected individuals, implementation and type of PPE, individual susceptibility, and activities that project airborne particles over greater distances in exhaled gas clouds) [92,93].

### 5.3. Exposure to COVID-19: Contact Tracing

**Figure 2:**
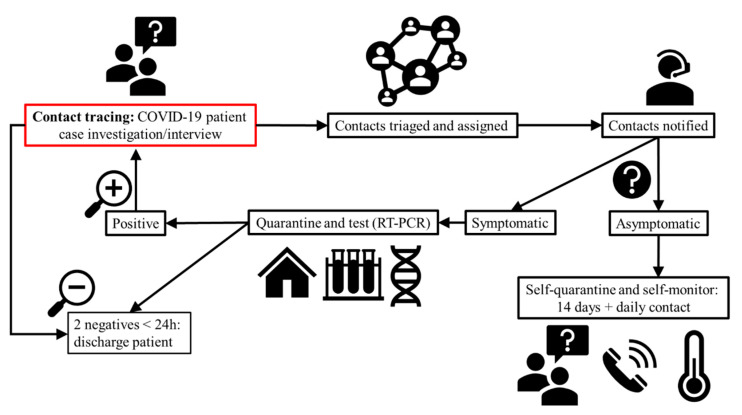
Contact tracing workflow for probable COVID-19 patients. Adapted from CDC [94,95] and WHO [71,96].

There are two scenarios in which quarantine should be implemented: (1) for travelers to prevent new infections through community transmission and; (2) for individuals with known infections and their close contacts [71]. To identify contacts of individuals with known infections, contact tracing should be implemented (Figure 2).

## 6. Conclusions

As reviewed in this paper, based on published information available at this time, there is no evidence to suggest that livestock animals and animal-derived food products are a source or transmission route of SARS-CoV-2. However, in relation to the food chain, the same cannot be asserted for food workers and the environment in which food workers work. Therefore, keeping all food supply chain workers healthy is an important challenge to maintain consumers’ confidence in food safety and to ensure food security on a global scale.

In order to ensure that the food supply chain remains intact, there is an urgent requirement for the food industry to introduce additional measures to protect food workers with an appropriate Food Safety Management System and effective detection tools for SARS-CoV-2. However, despite the unprecedented effort put forth by researchers to understand SARS-CoV-2 within a short period of time, there are still many unanswered questions about this novel virus, especially in regard to modes of transmission and factors that affect its stability and infectivity. Thus, research seeking to answer those questions is ongoing and is encouraged in order to restrict relevant crises in the future.

In our opinion, authorities should obtain detailed information about farmers and livestock producers (e.g., size and scale of operations, animal marketing flow, etc.) with the aim of connecting them with processors, retailers, and consumers. During a global health pandemic, producers could be very restricted in terms of marketing their livestock, and at the same time, consumers could be restricted in their accessibility to meat, milk, and eggs. Knowledge obtained through strengthened relationships between different sectors of the food supply chain could be associated with advanced communication technologies (e.g., Apps using big data and artificial intelligence), and these technologies could be used to collect real-time information in order to improve logistics, contact-tracing, and overall communication. The implementation of strong local, or short, food supply chains could also offer additional mechanisms to minimize risks related to food security and safety. Targeted assistance is particularly important to ensure that the benefits reach those most in need and guarantee food security for the most vulnerable populations of people.

## Figures and Tables

**Table 1:** Animal species that are proven to be susceptible to infection with SARS-CoV-2 (natural or/and experimental infection).

Species *	Infection (N or/and E)	Susceptibility (None/Low/High)	Clinical Signs	Transmission
Pigs	E	None	None	None
Poultry	E	None	None	None
Dogs	N and E	Low	Possible	Possible (suggested)
Cats	N and E	High	Possible **	Possible ***
Tigers and lions	N	High	Possible	Possible (between animals)
Ferrets	E	High	None or very mild	Possible ***
Minks	N	High	Possible	Possible ***; Mink to humans (suggested)
Fruit bats	E	High	No	Possible ***
Hamsters	E	High	Possible **	Possible ***
Macaques	E	High	Possible	Possible

* Poultry: chicken, ducks, and turkeys; Cats: domestic cat; Minks: *American minks* and *Neovison vison*; Fruit bats: Egyptian fruit bats *Rousettus aegyptiacus*; Hamsters: Golden Syrian hamsters; Macaques: *Macaca fascicularis* and *Macaca mulatta*. Infection N: natural; Infection E: experimental. ** None to very mild in some cases; *** Between same species. Adapted from Houssain et al. [5], Newman et al. [46], OIE [56], and Sit et al. [55].

**Table 2:** Disinfectants that are the most recommended for hands, surfaces, and textiles to prevent, control, and mitigate coronaviruses.

Places	Disinfectant	Concentration	Time	Ref.
Hands	Ethanol	60%	NS	[13]
Hands & Surfaces	Ethanol	70%	NS	[47,64,65,70,71] *
Surfaces	Alcohol-based disinfectants (ethanol, propan-2-ol, propan-1-ol)	70–80%	within 1 min	[53]
	Ethanol	62–71%	within 1 min	[56]
		60–85%	NS	[16] **
	Sodium hypochlorite (NaClO)	0.05%	NS	[16,47]
		1%	NS	[68,70] +
	Povidone-iodine	7.5%	NS	[70]
	Chloroxylenol	0.05%	NS	[70]
	Chlorhexidine	0.05%	NS	[70]
	Chlorhexidine digluconate	0.02%	NS	[56] ++
	Hydrogen peroxide	0.5%	within 1 min	[56]
	Benzalkonium chloride	0.05–0.2%	NS	[56] ++
		0.1%	NS	[70]
Cleaning equipmentused	Household soap or detergent	0.1%	within 1 min	[47] *+
	Sodium hypochlorite (NaClO)			[47,56] **
Textiles	Virucidal disinfectant		NS	[47]
	Hot-water cycle (90 °C) and regular laundry detergent			[47,71]
	Lower temperature cycle and bleach			[47]

NS: not specified. * For surfaces that cannot be cleaned with bleach, 70% ethanol can be used. ** can be used alongside regular household cleaning and disinfection. + Experiences with other CoVs such as SARS-CoV, MERS-CoV, or endemic human coronaviruses. ++ With less effectivity. *+: Second step of a cleaning procedure (area with suspected or confirmed case of COVID-19), after ventilation and before decontamination (Sodium hypochlorite).

**Table 3:** Time that SARS-CoV-2 and other coronaviruses can remain infectious (according to environment).

Source	Survival Time (up to)	Ref.
Dishes (dishwasher)	wash cycle *	[75]
Paper/Tissue paper	3 h	[68,70]
	28 days (20 ℃) *+	[76]
Copper surfaces	4 h **	[52]
Environmental +	3 h **	[52,56]
	16 h	[51]
Stool specimen (20 °C)	1 day ++	[68]
	2 days	[45]
Cardboard	24 h **	[52]
Wood	2 days	[70]
Cloth	1 day ++	[68]
	2 days	[70]
Stainless steel	3 days **	[52]
	7 days	[70]
	9 days ++	[56]
	28 days (20 ℃) *+	[76]
Plastic	2 days ++	[68]
	3 days **	[52]
	7 days	[70]
	9 days ++	[56]
Glass	4 days	[45,70]
	9 days ++	[56]
	28 days (20 ℃) *+	[76]
Mask		
Inner layer	7 days	[70]
Outer layer	More than 14 days	
Ambient		
70 °C	5 min	[16,70]
37 °C	from 1 to 2 days	[45,70]
from 20 to 25 °C	from 3 to 5 days	[45]
4 °C	>14 days (highly stable)	[45,48,70]
–20 °C	2 years	[1,16,48,75]
Sewage water		
20 °C	2 days ++	[56]
4 °C	14 days ++	
Respiratory specimen (throat and nasal)		
20 °C	5 days ++	[68]
4 °C	21 days ++	

* If temperatures higher than 60 °C are used; *+ specific laboratory conditions; ** albeit with significantly decreased titers; + in the air post-aerosolization (experimentally induced aerosols that do not reflect normal human cough conditions); ++ Experiences with other CoVs such as SARS-CoV, MERS-CoV, or endemic human coronaviruses.

**Table 4:** Recommended changes to prevent and control SARS-CoV-2 in facilities according to the following categories: structural, operational, and sociocultural.

Category	Challenges According to Practices and Sectors	Suggestions
Structural	Breaks, entering and exiting the facility (physical distancing)	—Adjust start and stop times (shifts), and outdoor breakrooms.
	Production line (Physical distancing)	—Physical barriers between workers.
	Worker’s health	—Screen all workers and visitors. —Contingency plan for isolation (workers who become ill).
Operational	Production line (physical distancing)	—Reduce the rate of food processing.
	Face covering recommendations	—Ensure adequate training and face coverings (agencies recommendation).
	Cleaning and disinfection routine	—Additional staff to sanitize “high touch” areas (e.g., handles, buttons, railings) more frequently. —Add hand sanitizers (dispensers and handwashing stations). —Implement touch-free time clocks.
	Processing rate for animals andcarcasses	—Mandatory face covering. —Good practice in donning protective equipment. —Touch-free time clocks. —Enhancing cleaning and disinfection.
Sociocultural	Employees that live in crowded, multigenerational settings	—Training about behaviors to limit the spread of the virus while at home.
	Employee transportation (to and from work)	—Add additional vehicles to shuttle routes —Face coverings

Adapted from Dyal et al. [18].

**Table 5:** Analytical methods available for SARS-CoV-2 detection according to samples (techniques), assays, and laboratory methods.

Sample Collect and/or Technique	Assays	Laboratory Method
Swab (NAAT)	RNA	RT-qPCR RT-LAMP NGS CRISPR ddPCR CBNAAT
Blood (Serological)	Ag/Ab	ELISA POC Lateral Flow CLIA
Image	Patient Chest	CT X-ray Ultrasound
Varied specimens	Virus particles	Electron microscopy

NAAT: nucleic acid amplification tests; RNA: ribonucleic acid; RT-qPCR: reverse transcriptase real-time polymerase chain reaction; RT-LAMP: loop-mediated isothermal amplification-based assay; NGS: next-generation sequencing; CRISPR: clustered regularly interspaced short palindromic repeats; ddPCR: droplet digital polymerase chain reaction; CBNAAT: cartridge-based nucleic acid amplification test; Ag/Ab: antigen/antibody; ELISA: enzyme-linked immunosorbent assay; POC: point-of-care; Lateral flow: handheld portable point-of-care; CLIA: chemiluminescence immunoassay; CT: computed tomography. Adapted from Rai et al. [82] and Pacarella et al. [83].
